# Supplementary material for: Understanding barriers to tuberculosis diagnosis and treatment completion in a low-resource setting: A mixed-methods study in the Kingdom of Lesotho
Source: PLoS One. 2023 May 11;18(5):e0285774. doi: 10.1371/journal.pone.0285774 (PMC10174523; doi:10.1371/journal.pone.0285774)
Supplement: S2 Checklist — (DOCX) [file pone.0285774.s002.docx]

**Good Reporting of A Mixed Methods Study (GRAMMS)**

| **Guideline** | **Section: page** |
| --- | --- |
| Describe the justification for using a mixed methods approach to the research question | Study design and objective: page 4 |
| Describe the design in terms of the purpose, priority and sequence of methods | Study design and objective: page 4 |
| Describe each method in terms of sampling, data collection and analysis | Quantitative data collection and analysis: pages 5-6  Qualitative data collection and analysis: pages 6-9 |
| Describe where integration has occurred, how it has occurred and who has participated in it | Qualitative data analysis (last paragraph): page 9 |
| Describe any limitation of one method associated with the presence of the other method | Discussion: page 28 |
| Describe any insights gained from mixing or integrating methods | Figure 4  Discussion: page 26 |

*O'Cathain A, Murphy E, Nicholl J. The quality of mixed methods studies in health services research. J Health Serv Res Policy. 2008;13(2):92-98.*
