# Supplementary material for: Understanding barriers to tuberculosis diagnosis and treatment completion in a low-resource setting: A mixed-methods study in the Kingdom of Lesotho
Source: PLoS One. 2023 May 11;18(5):e0285774. doi: 10.1371/journal.pone.0285774 (PMC10174523; doi:10.1371/journal.pone.0285774)
Supplement: S1 Table — (DOCX) [file pone.0285774.s003.docx]

**S1 Table.** **Numbers of individuals completing each step of the care cascade at two health facilities during March-August 2019**

|  | **Berea Hospital** | **Khubetsoana Health Center** |
| --- | --- | --- |
| People reporting symptoms | 218 | 292 |
| Sputum collected | 205 | 209 |
| Sputum sent to laboratory | 190 | 209 |
| Laboratory result recorded | 144 | 200 |
| Bacteriologically confirmed TB | 71 | 24 |
| Total TB diagnoses (all forms) | 90 | 44 |
| Treatment initiated | 90 | 43 |
| Treatment completed | 61 | 32 |
