# Supplementary material for: Understanding barriers to tuberculosis diagnosis and treatment completion in a low-resource setting: A mixed-methods study in the Kingdom of Lesotho
Source: PLoS One. 2023 May 11;18(5):e0285774. doi: 10.1371/journal.pone.0285774 (PMC10174523; doi:10.1371/journal.pone.0285774)
Supplement: Attachment — Submitted filename: PONE-D-22-29321R1 TB Africa mixed.docx [file pone.0285774.s006.docx]

Re: Understanding barriers to tuberculosis diagnosis and treatment completion in a lowresource setting: a mixed-methods study in the Kingdom of Lesotho

PONE-D-22-29321R1

Thank you for allowing us to review the authors' responses. The authors generally responded to all of my comments. Nonetheless, I have a few concerns:

**Abstract**

- Background and methods should be re-writing to avoid the redundancy of the information (“…to understand the barriers….”). Better to use the space to describe the analysis methods for both quantitative and qualitative designs.
- Findings: sample size should be mentioned before presenting the percentage. There is no information regarding the number of participants for the IDIs and FGDs.
- I am questioning the conclusion: "Providing social support such as food and transport support could prevent patients and families from experiencing financial hardship and help patients complete their treatment successfully”. The findings stated “food insecurity and high patient movement to search for jobs”. And the conclusion “…providing food and transport support… to complete their treatment”. So the transport barriers are related to “job seeking” and the conclusion is related to “the completion of the treatment”. Although it can be related indirectly, but not sure the authors can relate this based on a qualitative study.

**Reporting guidelines:**

Using a particular reporting guideline will improve the quality of the manuscripts. I agree with using COREQ as the reporting guidelines. However, COREQ is “Consolidated criteria for reporting qualitative research”; therefore, it is intended for a qualitative report. How about the quantitative findings? That’s why I suggested using GRAMMS, for example, or other reporting guidelines suitable for a mixed method.
