# Supplementary material for: Understanding barriers to tuberculosis diagnosis and treatment completion in a low-resource setting: A mixed-methods study in the Kingdom of Lesotho
Source: PLoS One. 2023 May 11;18(5):e0285774. doi: 10.1371/journal.pone.0285774 (PMC10174523; doi:10.1371/journal.pone.0285774)
Supplement: Attachment — Submitted filename: Response to reviewers.docx [file pone.0285774.s007.docx]

We have made the following changes in response to the reviewer’s comments.

**Comments about the abstract:**

**Comment**: Background and methods should be re-writing to avoid the redundancy of the information (“…to understand the barriers….”). Better to use the space to describe the analysis methods for both quantitative and qualitative designs.

**Response**: We have deleted the redundant phrase from the methods section and added details of the analysis: “We used a content analysis approach to analyze qualitative data and integrated quantitative and qualitative findings in a joint display.”

**Comment**: Findings: sample size should be mentioned before presenting the percentage. There is no information regarding the number of participants for the IDIs and FGDs.

**Response**: We have added all denominators for the quantitative results: “ During March-August, 2019, 218 clients at the hospital and 292 clients at the health center reported tuberculosis symptoms. The full diagnostic testing process was completed for 66% of clients at the hospital and 68% at the health center. Among clients who initiated tuberculosis treatment 68% (61/90) at the hospital and 74% (32/43) at the health center completed treatment.” We have added the number of participants in interviews and focus groups to the methods: “We conducted in-depth interviews and focus group discussions with 53 health workers and patients.”

**Comment**: I am questioning the conclusion: "Providing social support such as food and transport support could prevent patients and families from experiencing financial hardship and help patients complete their treatment successfully”. The findings stated “food insecurity and high patient movement to search for jobs”. And the conclusion “…providing food and transport support… to complete their treatment”. So the transport barriers are related to “job seeking” and the conclusion is related to “the completion of the treatment”. Although it can be related indirectly, but not sure the authors can relate this based on a qualitative study.

**Response**: We have revised the conclusion to eliminate the reference to transport: “Tuberculosis diagnosis could be improved through the effective decentralization of laboratory services at the health facility level, and treatment completion could be improved by providing food and other forms of social support to patients.”

**Comment about reporting guidelines:**

**Comment**: Using a particular reporting guideline will improve the quality of the manuscripts. I agree with using COREQ as the reporting guidelines. However, COREQ is “Consolidated criteria for reporting qualitative research”; therefore, it is intended for a qualitative report. How about the quantitative findings? That’s why I suggested using GRAMMS, for example, or other reporting guidelines suitable for a mixed method.

**Response**: We have uploaded the GRAMMS table in addition to the COREQ.
